# Metallothionein 2A with Antioxidant and Antitumor Activity Is Upregulated by Caffeic Acid Phenethyl Ester in Human Bladder Carcinoma Cells

**DOI:** 10.3390/antiox11081509

**Published:** 2022-08-01

**Authors:** Hsin-Ching Sung, Kang-Shuo Chang, Syue-Ting Chen, Shu-Yuan Hsu, Yu-Hsiang Lin, Chen-Pang Hou, Tsui-Hsia Feng, Ke-Hung Tsui, Horng-Heng Juang

**Affiliations:** 1 Department of Anatomy, College of Medicine, Chang Gung University, Kwei-Shan, Taoyuan 33302, Taiwan; hcs@mail.cgu.edu.tw (H.-C.S.); d000016684@cgu.edu.tw (K.-S.C.); stchen2021@mail.cgu.edu.tw (S.-T.C.); hsusy@mail.cgu.edu.tw (S.-Y.H.); 2 Graduate Institute of Biomedical Sciences, College of Medicine, Chang Gung University, Kwei-Shan, Taoyuan 33302, Taiwan; 3 Aesthetic Medical Center, Department of Dermatology, Chang Gung Memorial Hospital, Taoyuan 333, Taiwan; 4 Department of Urology, Chang Gung Memorial Hospital-Linkou, Kwei-Shan, Taoyuan 33302, Taiwan; laserep@mail.cgu.edu.tw (Y.-H.L.); glucose1979@cgmh.org.tw (C.-P.H.); 5 School of Nursing, College of Medicine, Chang Gung University, Kwei-Shan, Taoyuan 33302, Taiwan; thf@mail.cgu.edu.tw; 6 Department of Urology, Shuang Ho Hospital, New Taipei City 235041, Taiwan; 7 TMU Research Center of Urology and Kidney, Department of Medicine, College of Medicine, Taipei Medical University, Taipei 11031, Taiwan

**Keywords:** antioxidation, metallothionein 2A, HO-1, bladder cancer, apoptosis, ROS

## Abstract

Functions of metallothionein 2A (MT2A) in bladder cancer have not been extensively explored even though metallothioneins are regarded as modulators in several biological regulations including oxidation and cancerous development. We evaluated MT2A in bladder carcinoma cells in terms of the mechanisms of regulation and the underlying functions. MT2A overexpression not only downregulated endogenous ROS but also blocked ROS induced by H_2_O_2_. We used the annexin V-FITC apoptosis assay to determine the modulation of H_2_O_2_-induced cell apoptosis by MT2A expression. Results of immunoblot and reporter assays indicated that caffeic acid phenethyl ester (CAPE) treatment induced MT2A and heme oxygenase-1 (HO-1) expressions; moreover, the involvement of CAPE in either upregulation of the HO-1 expression or downregulation of endogenous ROS is MT2A dependent in bladder carcinoma cells. Knockdown of MT2A increased invasion and cell growth in vitro and in vivo, whereas ectopic overexpression of MT2A had the reverse effect in bladder carcinoma cells. Unlike bladder cancer tissues, the real-time reverse transcriptase-polymerase chain reaction (RT-qPCR) analysis showed a significant level of MT2A mRNA in the normal bladder tissues. Collectively, our results indicated that MT2A is acting as an antioxidant and also a tumor suppressor in human bladder carcinoma cells.

## 1. Introduction

Metallothioneins (MTs), initially identified from a horse kidney cortex in 1957, are a group of small cysteine-rich proteins [1]. Four main gene subfamilies of MTs (metallothionein 1 [*MT1*], metallothionein 2A [*MT2A*], metallothionein 3 [*MT3*], and metallothionein 4 [*MT4*]) are expressed in humans, and a cluster of *MT* genes is located on the chromosome 16q12-22 [2,3]. MTs were exploited as bio-environmental markers for predicting heavy metal contamination due to the nature of metal binding [4,5]. Further studies confirmed that MTs were involved in many biological processes including oxidation, cellular proliferation, differentiation, invasion, and carcinogenesis [6,7,8,9]. MT2A, found in various organs, tissues and culture cells, modulates a number of biological regulations, especially in oxidative stress [10]. Moreover, a study indicated that *MT2A* has tumor-suppressive activity through inhibiting nuclear transcription factor κB (NF-κB) signalling in gastric cancer patients [11]; however, the functions and expressions of *MT2A* in bladder cancer have not been thoroughly scrutinized.

Heme oxygenase-1 (HO-1) is the enzyme that determines the degradation rate of heme, and causes the release of carbon monoxide, ferrous iron, and biliverdin after heme is catalyzed [12]. HO-1 is a well-known inducible cytoprotective molecule that displays antioxidant, antiapoptotic, and anti-inflammatory effects. Therefore, HO-1 was considered as a potential candidate for investigating new therapeutic interventions [13,14]. In addition, the positive role of HO-1 in cancer progress was extensively demonstrated in several studies [15,16]. A study revealed that stable overexpression of human MT2A induced oxidative protection in a heart-derived cell line [17]; moreover, another study suggested that the expression of MT2A and HO-1 increased along with ROS during oxidative stress [18]. Thus, it is warranted to determine whether MT2A could control ROS through regulating HO-1 in bladder carcinoma cells.

Caffeic acid phenethyl ester (CAPE), an important active component of honey bee propolis, notably prevents translocation of the NF-κB subunits to the nucleus [19]. Our recent study revealed that CAPE has antitumor activity in bladder cancer [20]. Although the antioxidative activity of CAPE in the bladder has yet to be addressed, CAPE acting as a neuro-protector was well demonstrated by its antioxidative effects [21]. Nevertheless, the biological activity of CAPE on the expression of MT2A in bladder cancer has not yet been investigated. 

In this study, we aimed to elucidate the expression and function of MT2A in human bladder carcinoma cells and to examine the antioxidant and antitumor activity of MT2A in bladder cancer in vitro and in vivo.

## 2. Materials and Methods

### 2.1. Cell Culture and Chemicals

We obtained four bladder cancer cell lines (RT4, HT1376, TSGH-8301, and T24) from the Bioresource Collection and Research Center (BCRC; Hsinchu, Taiwan). The primary bladder epithelial cells (HBdEC; ATCC PCS-420-010), from the American Type Culture Collection (Manassas, VA, USA), were cultured as described previously [20]. We purchased characterized fetal bovine serum (FBS) from HyClone (SH30396.03; Logan, UT, USA), RPMI 1640 media from Invitrogen (Carlsbad, CA, USA), Matrigel from BD Biosciences (Bedford, MA, USA), CAPE from Selleckchem (Houston, TX, USA), and H_2_O_2_ from Sigma-Aldrich Co (St. Louis, MO, USA).

### 2.2. Expression Vectors and Overexpression

The full-length human MT2A expression vector (pCMV6-XL4-MT2A) was purchased from OriGene (SC125000; Rockville, MD, USA). The MT2A expression vector was transiently transfected into HT-1376 or TSGH-8301 cells, respectively, by electroporation as described previously [22]. We maintained the cells in a RPMI medium with 10% FBS and selected by G418 disulfate salt at 800 ng/mL (Sigma, St. Louis, MO, USA).

### 2.3. Knockdown of MT2A

We transduced T24 and HT1376 cells with control shRNA lentiviral particles-A (Sc-108080; T24_shCOL and HT_shCOL) and *MT2A* shRNA lentiviral particles (sc-93491-V; T24_shMT2A and HT_shMT2A), respectively. The transfected cells were selected by incubation with 4 μg/mL puromycin dihydrochloride for at least another 5 generations after 2 days of transduction.

### 2.4. Immunoblot Assay

Equal amounts (20 or 40 μg) of cell extracts were separated on a 10% or 12% SDS-PAGE gel, transferred and analyzed by the Western Lightning^™^ Plus Chemiluminescence detection system (Perkin Elmer, Inc., Waltham, MA, USA). The blotting membranes were probed using antibodies against HO-1 (Stressgen, Victoria, BC, Canada), MT2A (LS-C295358; LifeSpan BioSciences, Inc., Seattle, WA, USA), and β-actin (Millipore, Temecula, CA, USA). The LuminoGraph II (Atto Corporation, Tokyo, Japan) was used to record the band intensities, and the data were analyzed by Image J. 

### 2.5. Detection of ROS with Flow Cytometer

After treatment with CAPE, the cells were harvested with trypsin for 16 h. Cells were washed and suspended in a 10% FBS medium with 20 μM carboxy-H2DCFDA, and then left to incubate for 30 min at 37 °C. For studying the effect of H_2_O_2_, cells were trypsinized, washed and suspended in a 10% FBS medium with 20 μM carboxy-H2DCFDA, and then treated with or without 500 μM H_2_O_2_ for 30 min. Total ROS was analyzed using an Attune NxT acoustic focusing cytometer (Thermo Fisher Scientific Inc. Waltham, MA, USA) as described previously [22].

### 2.6. Real-Time Reverse Transcriptase-Polymerase Chain Reaction (RT-qPCR)

The TRIzol reagent (Ambion, Cartsbad, CA, USA) was used to extract the cells and isolate the total RNA. The superscript III preamplification system (Invitrogen, Carlsbad, CA, USA) was used to synthesize the cDNA. TaqMan^Tm^ gene expression master mix and PCR FAM dye-labeled probes for human *MT2A* (Hs01591333_g1). *HO-1* (Hs00157965_m1), *β-actin* (Hs01060665_g1), and *18S* (Hs03003631_g1) were from Applied Biosystems (Foster City, CA, USA). The CFX Connect Real-Time PCR system (Bio-Rad Laboratories, Foster city, CA, USA) was used to perform the quantitative real-time PCR as described previously [23]. The mean cycle threshold (Ct) values for the 18S or β-actin control probe were used in the normalization of target gene expression. All the reactions were carried out in triplicate, and each experiment was performed at least 3 times independently.

### 2.7. Annexin V-FITC Apoptosis Detection

Differentiation among early apoptotic cells (annexin V +/PI −), late apoptotic cells (annexin V +/PI +), and necrotic cells (annexin V −/PI +) was analyzed by flow cytometry with a annexin V-FITC/PI apoptosis detection kit (cat # K101-25; BioVision Inc., Milpitas, CA, USA). After treating the harvested pellets with annexin V-FITC and propidium iodide, apoptosis detection and quantification were performed. The Attune NxT acoustic focusing cytometer (Thermo Fisher Scientific Inc., Waltham, MA, USA) was used to count the fluorescence of cells. 

### 2.8. Cell Proliferation

Ki-67 was used as a cellular marker for proliferation to determine the proliferation rate or proliferation proportion. The cells were harvested and fixed with 3.7% paraformaldehyde for 10 min. Then, cells were permeabilized with 0.2% Triton X-100 for 10 min and were blocked in 1% bovine serum albumin (A7906, Sigma–Aldrich Co., St. Louis, MO, USA) in phosphate-buffered saline (PBS) solution thereafter. After an hour of incubation, the cells were stained with Ki-67 antibody conjugated PE (Ki67-PE) for 30 min in the dark. Flow cytometry was used to measure the proportion of Ki-67-positive cells by an Attune NxT acoustic focusing cytometer (Thermo Fisher Scientific Inc., Waltham, MA, USA).

### 2.9. Matrigel Invasion Assay

The invasion ability of the cells was determined with an in vitro Matrigel invasion assay. After 24 h (for T24 and TSGH-8301 cells) or 48 h (for HT1376 cells) of cell migration to the Matrigel-coated transmembrane, we fixed it with 4% paraformaldehyde and stained it with 0.1% crystal violet solution for 10 min. The quantity of invaded cells in the Matrigel was microscopically (IX71, Olympus, Tokyo, Japan) analyzed using Image J as described previously [23].

### 2.10. Report Vector Constructs and Reporter Assays

As described previously, we constructed a DNA fragment containing a 5’-flanking region (−4333 to +22) of the *HO-1* gene from a BAC clone (CTA-286B10; Wellcome Trust Sanger Institute, Cambridge, UK) and cloned it into the pGL3-Basic reporter vector (Promega BioScience, San Luis Obispo, CA, USA) [22]. One 3397 bp of DNA fragment containing the enhancer/promoter of the *MT2A* gene was isolated from the BAC clone (RP11-249C24, Invitrogen, Carlsbad, CA, USA) and cloned into the pGEM3 vector at *Sac I* sites. Using this target DNA fragment, the human MT2A promoter/enhancer DNA fragment (+9 to −1733) was synthesized by PCR with primers (5′-AGATCTGGGACTTGGAGGAGGCGTGG-3′ and 5′-TAATACGACTCACTATAGGG-3′). The DNA fragment was digested and cloned into the pGL3-basic luciferase reporter vector (Promega Biosciences San Luis Obispo, CA, USA) at the *Sac I* and *Bam HI* sites. Cells were plated onto a 24-well plate at 1 × 10^4^ cells/well for one day prior to transfection. Cells were transiently transfected using the X-tremeGene HP DNA transfection reagent (Roche Diagnostics GmbH, Mannheim, Germany) with reporter vectors and expression vectors as indicated for 4 h. Then, the cells were cultured in a RPMI 1640 medium with 10% FBS after another 48 h of incubation with/without various concentrations of CAPE as indicated. The luciferase activity was determined in relative light unit using a Synergy H1 microplate reader (BioTek, Beijing, China) and was adjusted by the concentration of protein in the whole-cell extract, which was measured using a bicinchoninic acid protein assay kit (Pierce Protein Research, Rockford, IL, USA).

### 2.11. Xenograft Animal Study

The 4-week-old male nude mice (BALB/cAnN-Foxn1) were purchased from the animal center of the Ministry of Science and Technology in Taiwan. HT_shCOL and HT_shMT2A cells were detached from the cell flask through a treatment with Gibco Versene solution (Life Technologies, Grand Island, NY, USA), and washed with a RPMI1640 medium with 10% FBS. Animals were randomized into two groups and were anesthetized intraperitoneally. The equal amounts of cells (6 × 10^6^/100 μL PBS) were subcutaneously injected on the lateral back wall of each mouse at day 0. Tumor volume was measured every 3 days using vernier calipers and presented using the following formula: volume = π/6 × larger diameter × (smaller diameter)^2^ as described previously [23]. The tumor mRNA and protein levels of MT2A and HO-1 from HT_shCOL and HT_shMT2A cells were examined by immunoblot and RT-qPCR assays as described above after mice were sacrificed.

### 2.12. Tissue Collection and Analysis

The pair specimens of cancer and adjacent mucosal tissues were collected from 26 bladder cancer patients at the Department of Urology, Chang Gung Memorial Hospital-Linkou (Tao-Yuan, Taiwan). All 26 patients had undergone radical cystectomy with pelvic lymph node dissection and were all at least at clinical stage T2. After removing the bladder, the surgeon excised a small fragment of the visible tumor mass and the surrounding normal mucosal tissues. The differences in the respective specimens from the urothelial carcinoma and normal bladder tissues were verified by the attending pathologist. After radical cystectomy, all bladder samples were washed with PBS and frozen in a liquid nitrogen tank immediately. Tissues were extracted with TRIzol reagent, and then the cDNA was synthesized and analyzed by RT-qPCR as described above. 

### 2.13. Statistical Analysis

Results are expressed as the mean ± standard error (S.E.) of at least three independent experiments. Statistical significance (*, *p* < 0.05; **, *p* < 0.01) was determined by Student’s *t*-test and one-way ANOVA. The application of post hoc analysis was to correct for multiple comparisons using SigmaStat software for Windows version 2.03 (SPSS Inc., Chicago, IL USA).

## 3. Results

### 3.1. Expressions of MT2A in Bladder Tissues and Carcinoma Cells

**Figure 1:**
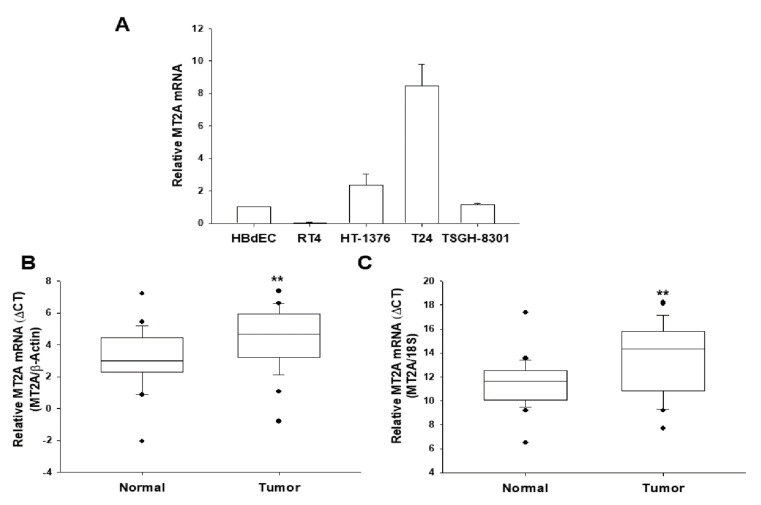
Expression of MT2A in human bladder tissues and cells. (**A**) The expression of MT2A in bladder cells was determined through RT-qPCR assays (±SE, *n* = 3). The numbers of mRNA levels indicated the ratio of MT2A/β-Actin in relation to HBdEC cells. RT-qPCR assays using β-Actin (**B**) or 18S (**C**), respectively, as the internal control, was conducted to quantitatively analyze the MT2A mRNA levels in paired bladder cancer and normal tissues. The comparison of the MT2A expressions in normal bladder and cancer tissues was examined by box plot analysis (*n* = 26). ** *p* < 0.01.

The levels of MT2A mRNA in cultured bladder cell lines (HBdEC, RT4, HT1376, T24, and TSGH-8301) were evaluated. The RT-qPCR assays showed T24 cells with the highest and RT4 cells with the lowest MT2A levels among the four bladder cancer cell lines as compared to normal bladder epithelial cells, HBdEC (Figure 1A). The RT-qPCR analysis of paired human bladder tissues (*n* = 26; 5 low grade and 21 high grade) showed that the mean ΔΔCt was 1.3454 ± 0.3784 when using β-actin as the internal control (Figure 1B) and 2.0738 ± 0.6027 when using 18S as the internal control (Figure 1C) in normal and bladder cancer tissues, indicating that normal bladder tissues contained significantly higher MT2A mRNA levels (*p* = 0.0014 and *p* = 0.002; when using β-actin or 18S as the internal control, respectively). 

### 3.2. MT2A Knockdown Downregulates Heme Oxygenase-1 (HO-1) but Upregulates Reactive Oxygen Species (ROS) in Bladder Carcinoma Cells

**Figure 2:**
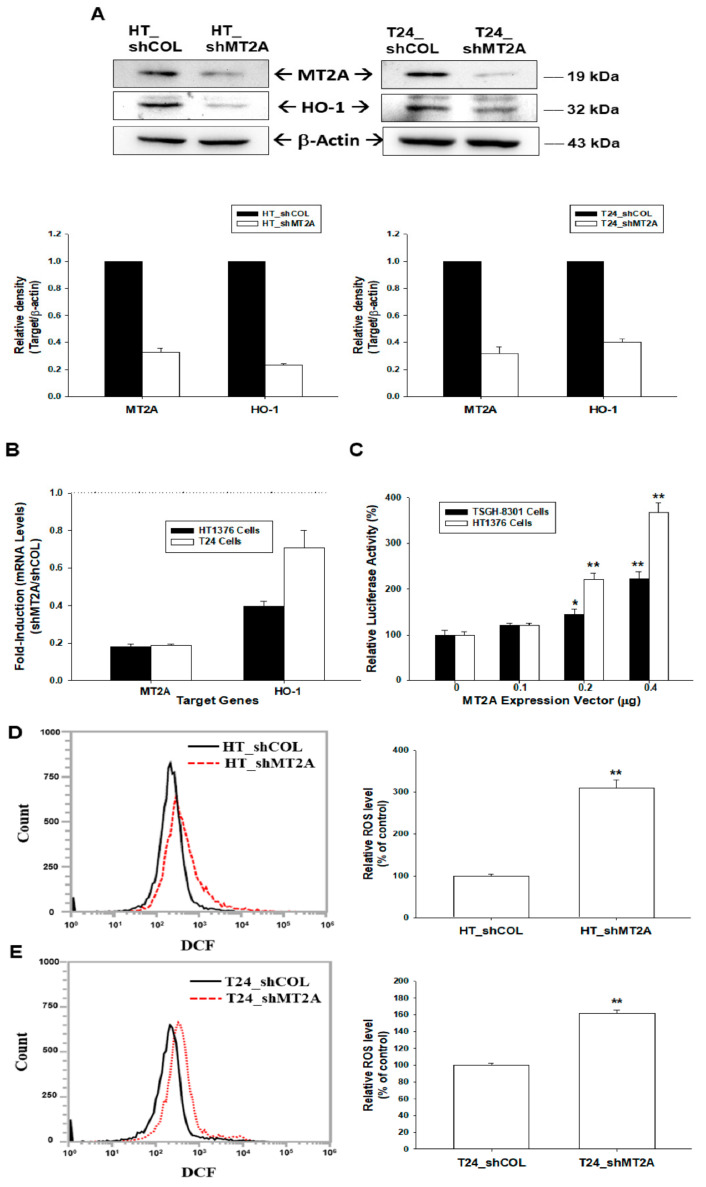
MT2A modulates the heme oxygenase-1 and endogenous reactive oxygen species in bladder carcinoma cells. (**A**) Protein levels of MT2A and HO-1 after knockdown of MT2A in HT1376 (left) and T24 (right) cells were examined by immunoblot assays. The quantitative data are presented as the intensity of the protein bands of the target proteins/β-actin relative to the mock-knockdown cells (bottom). (**B**) Relative fold-induction mRNA levels of MT2A and HO-1 in HT_shMT2A and T24_shMT2A cells compared with HT_shCOL and T24_shCOL cells, respectively, determined by using RT-qPCR assays. (**C**) Relative luciferase activity of HO-1 reporter vector after co-transfection with various concentrations of MT2A expression vector in TSGH-8301(black bars) and HT1376 (white bars) cells (±SE, *n* = 6). The endogenous ROS levels in HT_shCOL, HT_shMT2A (**D**), T24_shCOL, and T24_shMT2A (**E**) cells were measured by flow cytometry. * *p* < 0.05; ** *p* < 0.01.

In immunoblot assays of HT1376 and T24 cells, the knockdown of MT2A downregulated HO-1 protein levels was found (Figure 2A). RT-qPCR also exhibited similar results (Figure 2B). Further reporter assays revealed that transient overexpression of MT2A induced promoter activity of the human HO-1 gene dose-dependently (Figure 2C). We continued to determine the antioxidant response of MT2A in human bladder carcinoma cells. The flow cytometry assays indicated that knockdown of MT2A in HT1376 (Figure 2D) or T24 (Figure 2E) cells induced a 3.09- and 1.61-fold increase in endogenous ROS.

### 3.3. Expression of MT2A Modulates H_2_O_2_-Induced Cell Apoptosis in Bladder Carcinoma Cells

**Figure 3:**
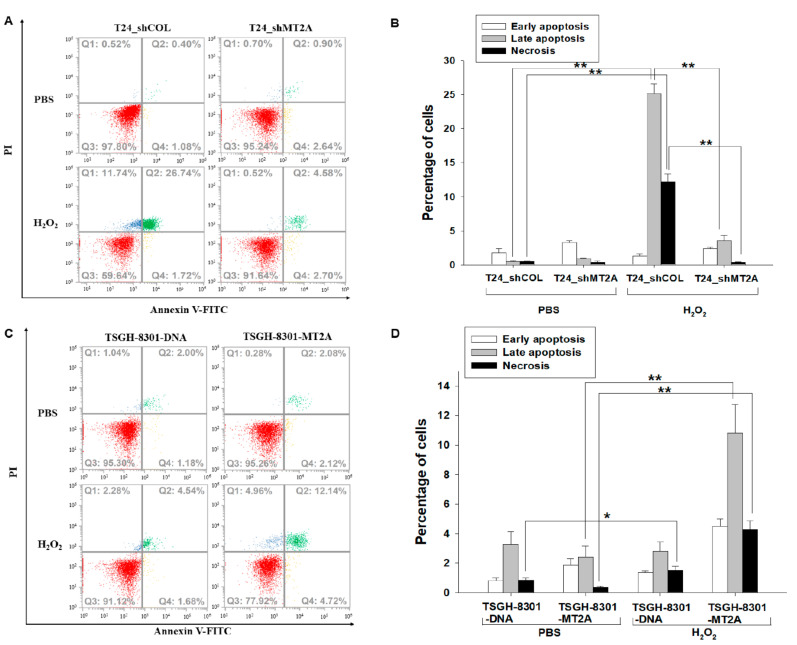
MT2A enhances H_2_O_2_ treatment-induced cell apoptosis in bladder carcinoma cells. Cell apoptosis was determined by the association of annexin V-FITC with PI staining. The fluorescence intensity of mock-knockdown (T24_shCOL) and MT2A knockdown (T24_shMT2A) cells after 500 μM of H_2_O_2_ treatment for 3 h was determined by flow cytometry (**A**). The quantitative data were presented as the percentage of early apoptosis, late apoptosis and necrosis of cells after treatments as indicated in T24 cells (**B**). Flow cytometry was used to determine the fluorescence intensity of mock-overexpressed TSGH-8301 (TSGH-8301_DNA) and MT2A-overexpresssed TSGH-8301 (TSGH-8301-MT2A) after 500 μM of H_2_O_2_ treatment for 3 h (**C**). The quantitative data were presented as the percentage of the early apoptosis, late apoptosis and necrosis of cells after treatments as indicated in TSGH-8301 cells (**D**). Fluorescence intensity of the annexin V-FITC is plotted on the *x*-axis, and the PI is plotted on the *y*-axis. * *p* < 0.05; ** *p* < 0.01.

We distinguished early apoptotic, late apoptotic, and necrotic cells by annexin V-FITC in conjunction with PI staining. The fluorescence intensity of annexin V-FITC and PI in T24_shCOL and T24_shMT2A cells after 500 μM of H_2_O_2_ treatment for 3 h displayed that MT2A knockdown in T24 cells (T24_shMT2A) was significantly resistant to H_2_O_2_-induced cell apoptosis compared to the mock-knockdown (T24_shCOL) cells (Figure 3A). Quantitative analysis indicated that T24_shCOL cells, as well as T24_shMT2A cells, with H_2_O_2_ treatment did not significantly affect the percentage of cells in early apoptosis. However, MT2A knockdown significantly resisted H_2_O_2_ treatment-induced late apoptosis (25.17% vs. 3.55%) and necrosis (12.17% vs. 0.38%) of T24_shCOL cells in comparison to T24_shMT2A cells (Figure 3B). Inversely, the ectopic overexpression of MT2A in TSGH-8301 cells significantly enhanced H_2_O_2_-induced cell apoptosis by comparing mock-overexpression TSGH-8301 cells (Figure 3C). Quantitative analysis further confirmed that the overexpression of MT2A significantly increased H_2_O_2_ treatment-induced early apoptosis, late apoptosis, and necrosis (1.35% vs. 4.52%, 2.80% vs. 10.81%, and 1.51% vs. 4.29%, respectively) of TSGH-8301-DNA cells in comparison to TSGH-8301-MT2A cells (Figure 3D).

### 3.4. Overexpression of MT2A Alleviates H_2_O_2_-Induced ROS in Bladder Carcinoma HT1376 Cells

**Figure 4:**
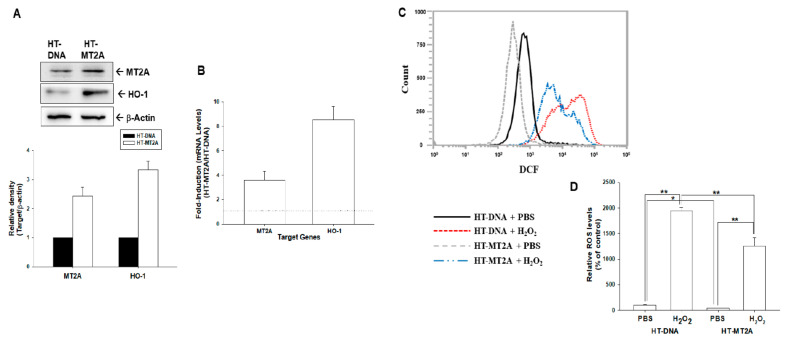
MT2A downregulated H_2_O_2_-induced ROS in bladder carcinoma HT1376 cells. (**A**) Protein levels of MT2A and HO-1 after overexpression of MT2A in HT1376 cells were examined by immunoblot assays (top). The quantitative data were presented as the intensity of the protein bands of the target proteins/β-actin relative to the mock-overexpressed cells (bottom). (**B**) Relative fold-induction of mRNA levels of MT2A and HO-1 in HT-MT2A cells compared with HT-DNA cells was evaluated by RT-qPCR assays. ROS levels (**C**) and quantitative data (**D**) of HT-DNA and HT-MT2A cells after treatment with or without H_2_O_2_ were measured by flow cytometry. * *p* < 0.05; ** *p* < 0.01.

We determined the antioxidant response of overexpression of MT2A in human bladder carcinoma HT1376 cells. The immunoblot assays exhibited that overexpression of MT2A increased HO-1 protein levels in HT1376 cells (Figure 4A). This finding was similar in RT-qPCR assays (Figure 4B). The flow cytometry assays indicated that generation of ROS was stimulated by 500 μM of H_2_O_2_ treatment in mock-overexpressed HT1376 (HT-DNA) cells; on the other hand, ectopic overexpression of MT2A depressed H_2_O_2_-induced ROS generation in HT-MT2A cells (Figure 4C). Quantitative analysis is shown in Figure 4D.

### 3.5. Caffeic Acid Phenethyl Ester Induces MT2A Expression to Downreguate Endogenous ROS in Bladder Carcinoma Cells

**Figure 5:**
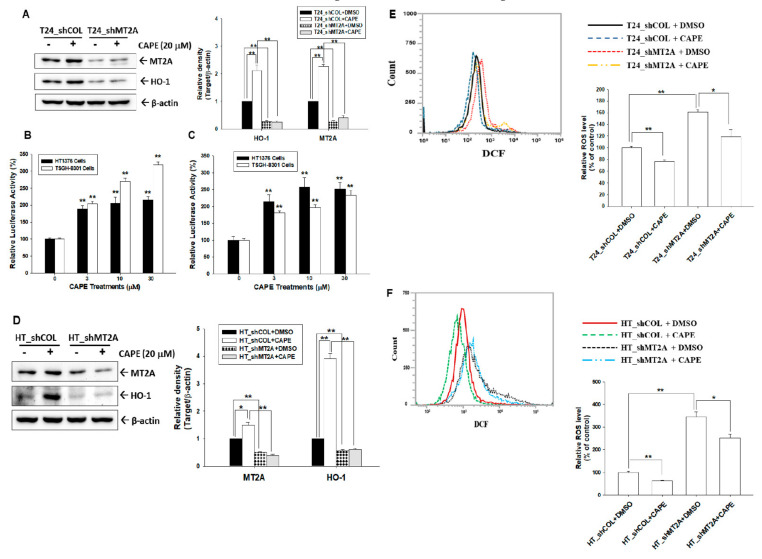
Caffeic acid phenethyl ester induces MT2A and HO-1 expressions to downregulate endogenous ROS in bladder carcinoma cells. (**A**) Protein levels of MT2A and HO-1 after CAPE treatments in T24_shCOL and T24_shMT2A cells were determined by immunoblot assays (left). The presented quantitative data were the intensity of the protein bands of the target proteins/β-actin relative to the mock-knockdown cells (right). The relative luciferase activity of HT1376 (black bars) and TSGH-8301 (white bars) cells were transfected with the human MT2A (**B**) or HO-1 (**C**) reporter vectors and treated with various concentrations of CAPE. Presented data were the mean percentage (±SE, *n* = 6) compared with the vehicle-treated cells. (**D**) Protein levels of MT2A and HO-1 after CAPE treatments in HT_shCOL and HT_shMT2A cells were examined by immunoblot assays (left). The quantitative values presented were the intensity of the protein bands of the target proteins/β-actin relative to the mock-overexpressed cells (right). (**E**) Endogenous ROS levels (left) and quantitative data (right) of T24_shCOL and T24_shMT2A after treatment with or without CAPE were determined by flow cytometry. (**F**) Endogenous ROS levels (left) and quantitative data (right) of HT_shCOL and HOT_shMT2A cells after treatment with or without CAPE were measured by flow cytometry. * *p* < 0.05; ** *p* < 0.01.

Immunoblot assays revealed that CAPE treatment (20 μM) upregulated MT2A and HO-1 expressions in bladder carcinoma T24 cells; moreover, knockdown of MT2A attenuated the activation of CAPE on HO-1 protein levels (Figure 5A, left). Results of quantitative analyses from three independent experiments were presented in Figure 5A (right). Reporter assays using the MT2A and HO-1 reporter vectors with a specific human MT2A and HO-1 promoters, respectively, revealed that CAPE induced MT2A (Figure 5B) and HO-1 (Figure 5C) reporter activities dose dependently in bladder carcinoma HT1376 and TSGH-8301 cells. Similar results were presented in bladder carcinoma HT1376 cells, in which CAPE treatment upregulated MT2A and HO-1 expressions and MT2A knockdown attenuated the activation of CAPE on HO-1 protein levels (Figure 5E). Further flow cytometry assays indicated that endogenous ROS was upregulated when MT2A was knocked down in bladder carcinoma cells. CAPE downregulated endogenous ROS in T24 and HT1376 cells, while knockdown of MT2A attenuated the CAPE activation of endogenous ROS in T24 (Figure 5D) and HT1376 (Figure 5F) cells.

### 3.6. Effect of MT2A on In Vitro Cellular Proliferation and Invasion in Bladder Carcinoma Cells

**Figure 6:**
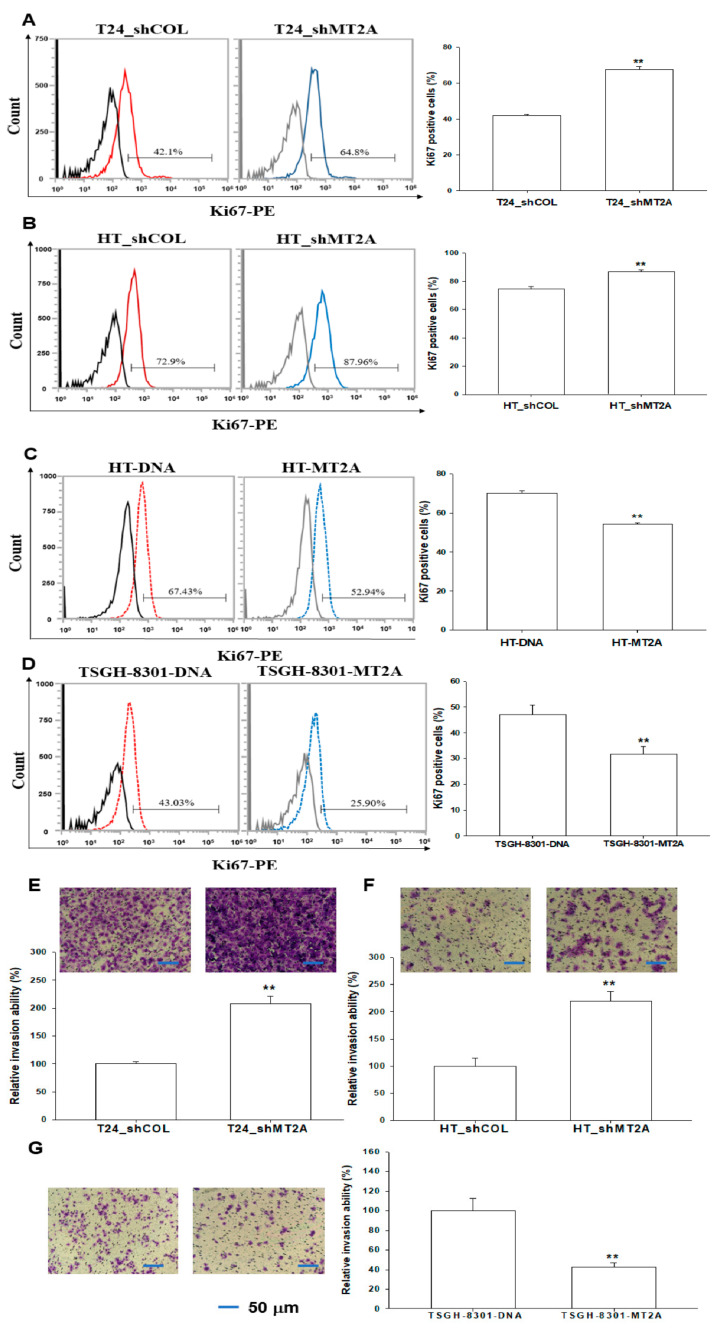
Modulating effect of MT2A on cellular proliferation and invasion in bladder carcinoma cells. The abilities of cellular proliferation in T24_shCOL, T24_shMT2A (**A**), HT_shCOL, HT_shMT2A (**B**), HT-DNA, HT-MT2A (**C**), TSGH-8310, and TSGH-8301-MT2A cells (**D**) were measured by flow cytometry using a Ki67 flow cytometry kit (±SE, *n* = 4). The cellular invasion ability was determined by in vitro Matrigel invasion assays. Data are presented as the mean percentage (±SE; *n* = 3) in relation to the (**E**) T24_shCOL, (**F**) HT_shCOL, or (**G**) TSGH-8301-DNA cells. The scale bar is 50 μm. ** *p* < 0.01.

Ki67 flow cytometry showed that MT2A knockdown in T24 and HT1376 cells (T24_shMT2A and HT_shMT2A) increased the numbers of cells with Ki67 staining by 15% and 12% compared to the mock-transduced (T24_shCOL and HT_shCOL) cells, respectively (Figure 6A,B). However, the ectopic overexpression of MT2A MT2A in HT1376 and TSGH-8301 cells exhibited the opposite results. The Ki67 staining proliferation assays indicated that overexpression of MT2A in HT1376 and TSGH-8301 cells (HT-MT2A and TSGH-8301-MT2A) decreased the amount of cells with Ki67 staining by 16% and 15% compared with mock-overexpression (HT-DNA and TSGH-8301-DNA) cells, respectively (Figure 6C,D). Moreover, the Matrigel invasion assays showed a significant increase in cellular invasion in the MT2A knockdown of T24 (Figure 6E) and HT1376 cells (Figure 6F). In contrast, overexpression of MT2A significantly inhibited cell invasion in TSGH-8301 cells (Figure 6G).

### 3.7. Knockdown of MT2A Enhances Tumorigenesis of HT1376 Cells

**Figure 7:**
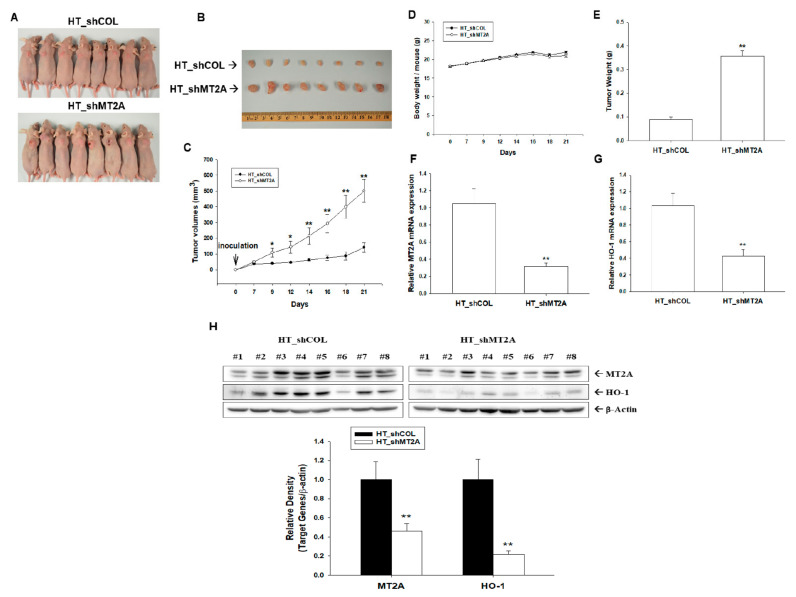
Modulating effect of MT2A on tumor growth of bladder carcinoma HT1376 cells. Four-week-old male athymic nude mice were divided randomly into two groups. (**A**) HT_shCOL and HT_shMT2A cells (6 × 10^6^) were injected subcutaneously in the dorsal area of the mice (*n* = 8). (**B**) Tumors from HT_shCOL and HT_shMT2A cells were recorded after mice were sacrificed. The volumes of tumor (**C**) and body weight (**D**) were measured every 2–3 days during a period of 21 days. The (**E**) tumor weight (±SE; *n* = 8), mRNA levels (±SE; *n* = 8) of MT2A (**F**) and HO-1 (**G**), and (**H**) protein levels of MT2A and HO-1 (±SE; *n* = 8) of the tumors from HT_shCOL and HT_shMT2A cells were recorded after mice were sacrificed. * *p* < 0.05; ** *p* < 0.01.

Since we determined MT2A as an antitumor gene (Figure 1) and downregulated cell proliferation in vitro (Figure 6), we continued to assess the effect of MT2A on tumor growth in vivo. We xenografted HT_shCOL and HT_shMT2A cells into the male nude mice (BALB/cAnN-Foxn1). The measurement of body weight and tumor volume was performed twice per week during the experimental period. On the 21st day after inoculation, the mice were sacrificed and the tumors were collected (Figure 7A,B). From the results, the tumors in the HT_shCOL group grew slowly compared to the tumors in the HT_shMT2A group (141.32 ± 30.60 vs. 501.15 ± 71.64 mm^3^) during 21 days of the experimental period (Figure 7C). However, the average body weight of the HT_shMT2A group was not significantly different from the vehicle-treated group (Figure 7D). The average tumor weight in the HT_shMT2A group was heavier than in the HT_shCOL group (0.09 ± 0.01 vs. 0.36 ± 0.02 g; Figure 7E). The mRNA levels of MT2A (Figure 7F) and HO-1 (Figure 7G) were downregulated by approximately 70% and 58%, respectively, in the xenograft tumors derived from HT_shMT2A cells compared to those derived from HT_shCOL cells. Further immunoblot assays confirmed that not only was MT2A knocked down but that the protein level of HO-1 also decreased in the xenograft tumors derived from HT_shMT2A cells (Figure 7H). Collectively, the above results suggest that MT2A knockdown in highly metastatic HT1376 cells significantly enhances the growth of tumors in the xenograft mice model.

## 4. Discussion

Although MTs have been recognized as the modulators in certain biological processes and shown to be common in many biological organisms, systematic examination of MTs in relation to the biochemical research concerning expression, regulatory mechanisms, and function of MTs in human diseases is divergent [3,6,8,24]. Recent studies have suggested that *MT3* is an arsenic-upregulated oncogene for the tumor growth and invasion of prostate and bladder carcinoma cells [9,25]. RT-qPCR assays in Figure 1A revealed the highest levels of *MT2A* in T24 cells as compared to the other three bladder cell lines (RT4, HT1376, and TSGH-8301), which is contrary to the results of an earlier study in MT3 [9], suggesting that MT2A and MT3 expressions in bladder carcinoma cells are due to the cellular type but not related to the extent of neoplasia in vitro. RT4 cells are bladder papillary tumor cells, and HT1376, TSGH-8301, and T24 cells are urothelial carcinoma cells. These cells express quite different expressions of p53 and PTEN and have divergent tumorigenic capability unrelated to the grades of original derived from explants tumor [9]. Prior in vitro studies have indicated that expressions of target genes in such bladder cell lines may not be due to the in vivo neoplasia [23]. Further RT-qPCR analysis of paired human bladder tissues indicated that MT2A mRNA expression is significantly higher in normal bladder tissues than bladder cancer tissues, verifying the role of MT2A on tumor suppression (Figure 1B, 1C). These results are consistent with one early study which suggested that MT2A might play a tumor-suppressive role in gastric cancer [11]. Previous studies have indicated that MT1E, MT1F and MT1M are antitumor genes in malignant melanoma, colon carcinoma cells, and hepatocellular carcinoma HepB3, respectively [26,27,28]. This is the first report to conclude that MT2A is the antitumor gene of bladder, and even the present data may not be adequate to correlate MT2A mRNA expression with the clinical-pathological characteristics of patients due to the small sample size. Future investigation engaging a larger sample size and verifying the protein levels are warranted. 

MTs are known to participate in an array of protective stress responses by protecting cells from exposure to oxidants and electrophiles [29]. Prior studies have indicated that MTs, because of the cysteine thiolate groups, scavenge hydroxyl radicals in vitro and in vivo [30,31]. In particular, MT2A was well evaluated as a ROS modulator [10]. However, the antioxidative effect of MT2A in bladder carcinoma cells is yet to be determined. In the present study, our results indicated that knockdown of MT2A induced endogenous ROS (Figure 2); in addition, ectopic overexpression of MT2A MT2A blocked H_2_O_2_-induced ROS generation in bladder carcinoma cells (Figure 4). These results are in agreement with a study revealing that stable overexpression of MT2A increased oxidative protection in a human heart-derived cell line [17]. Interestingly, reports also suggested that MTs may help cancer cells to survive by inhibiting apoptosis or suppressing cell growth via augmenting apoptosis in different types of cells [27,28,32]. The results of the present study indicated that knockdown of MT2A significantly resisted H_2_O_2_ treatment-induced cell apoptosis, while ectopic overexpression of MT2A significantly enhanced cell apoptosis induced by H_2_O_2_ in bladder carcinoma cells (Figure 3).

Propolis (bee glue) is a natural compound produced by honeybees and was widely used in traditional medicine. CAPE, the main bioactive component of propolis, has exhibited several biomedical properties including anticancer and antioxidant activity [33]. Our recent study revealed that CAPE induces growth differentiation factor 15 to occlude the growth of bladder carcinoma cells in vitro and in vivo [20]. Recent reports indicated that CAPE reduces oxidation stress of various cells in vitro and in vivo, which may be caused by the enhancement of HO-1 expression [34,35]. However, no report has yet to determine the antioxidant activity of CAPE on bladder carcinoma cells. In the present study, our results demonstrated that CAPE treatment upregulated MT2A and HO-1 expressions to alleviate endogenous ROS in bladder carcinoma cells which is consistent with other studies in HL-60 and H9c2 cells [36,37]. The results of CAPE induced HO-1 expression in the present study is consistent with other studies in murine bone marrow macrophages and human gingival fibroblast [34]. Moreover, the function of HO-1 is well known as the antioxidant in various cell types of human [38]. Our previous studies further indicated that HO-1 has antioxidative activity in the hepatoma cells [22]. Moreover, an early study has indicated that MT2A and HO-1 are increased along with the stimulation of gallium nitrate in human lymphoma cells [18]. The antioxidative activity of HO-1 and its cooperation with MT2A in bladder carcinoma cells have yet to be disclosed. One of the MTs, MT3, was found to upregulate HO-1 under the stimulation of 6-hydroxydopamine-induced oxidative stress in dopaminergic SH-SY5Y cells [39]. Collectively, these results are the first study indicating that MT2A modulated HO-1 expression (Figure 2 and Figure 4); moreover, CAPE induced MT2A and HO-1 to alleviate endogenous ROS and the activation of CAPE on HO-1 is dependent on MT2A in bladder carcinoma cells (Figure 5).

MTs have been found to be associated with human cancers [40,41]. Reports have illustrated that MT2A is the modulator of cell proliferation and apoptosis in vitro and in vivo [42,43]. Results from in situ, in vitro, and in vivo studies have suggested that MT2A is an antitumor gene in gastric and colorectal cancers [11,43,44]. The results of our RT-qPCR assays showed significant differences in MT2A mRNA levels between paired cancer tissues and normal tissues when using β-actin or 18S as the internal control (Figure 1). We continued to evaluate the MT2A antitumor activity of bladder carcinoma cells in vitro and in vivo. Results of in vitro Ki67 proliferation and Matrigel invasion assays showed that MT2A knockdown bladder carcinoma cells grew faster and increased cell invasion more than the mock-knockdown bladder carcinoma cells, while MT2A-overexpressed bladder carcinoma cells grew slower than the mock-transfected bladder carcinoma cells (Figure 6). The xenograft study also presented that mock-knockdown bladder carcinoma HT1376 cells grew slower than the MT2A knockdown bladder carcinoma HT1376 cells in vivo (Figure 7). The results are different from our early studies in urogenital carcinoma cells, exhibiting MT3-induced cell growth and tumorigenesis in vitro and in vivo [9,25]. As far as we are concerned, this is the first study providing laboratory values to demonstrate the tumor-suppressive role of MT2A in human bladder carcinoma cells.

## 5. Conclusions

Our results confirmed that expression of MT2A occurs with that of HO-1, which not only downregulates endogenous ROS but also blocks ROS generation induced by H_2_O_2_. Expression of MT2A modulates H_2_O_2_-induced cell apoptosis. CAPE induces MT2A and HO-1 expressions to downregulate endogenous ROS in bladder carcinoma cells; however, the induction of CAPE on HO-1 expression is MT2A dependent. Ectopic overexpression of MT2A not only attenuates cell invasion but also attenuates cell growth in vitro and in vivo. Our findings suggest that MT2A is an antioxidative as well as an antitumor gene in bladder carcinoma cells. Further work is needed to confirm the application of MT2A and related inducers against tumorigenesis and oxidative consequences of bladder cancer.

## Data Availability

All of the data are presented in this article.
